# Editorial: Effects of Probiotics and Prebiotics on Gut Pathogens and Toxins

**DOI:** 10.3389/fmicb.2022.856779

**Published:** 2022-02-28

**Authors:** Qing Kong, Tongjie Liu, Hang Xiao

**Affiliations:** ^1^School of Food Science and Engineering, Ocean University of China, Qingdao, China; ^2^Department of Food Science, University of Massachusetts Amherst, Amherst, MA, United States

**Keywords:** probiotics, prebiotics, mechanism, gut health, microbiota

Probiotics are “live microorganisms that, when administered in adequate amounts, confer a health benefit on the host” (Hill et al., 2014). Specific strains especially those from *Lactobacillus* and *Bifidobacterium* have been introduced as probiotics and have been consumed throughout the world. Probiotics can regulate the microecological balance of the digestive tract, inhibit the growth and adhesion of pathogenic bacteria, and ameliorate inflammation, thereby supporting a healthy digestive tract. Prebiotics are substrates that are selectively utilized by host microorganisms conferring a health benefit and they can also defense against pathogens and modulate gut microbiota (Gibson et al., 2017). Use of some probiotics and prebiotics is endorsed by robust efficacy evaluations. However, our knowledge on their mechanisms is mainly based on research using *in vitro*, animal or *ex vivo* human models and not all mechanisms have been confirmed in humans (Pujari and Banerjee, 2021).

Probiotics and prebiotics function in the intestine *via* crosstalk with the host and the commensal bacteria in certain ways, which, till now, are not fully understood regarding the specific molecules directly conferring the health benefits, the host targets of these molecules and the signal transduction pathways. The current understanding of the interactions between probiotics, prebiotics, microbiota, and pathogens in the gut is insufficient, which limits the application of probiotics and prebiotics (Cunningham et al., 2021).

We received 14 submissions in this Research Topic, and finally seven research articles and one review passed the strict peer review process and were accepted for publication. The studies in this Research Topic show that the function of probiotics occurs mainly in the intestine. Rodríguez-Sorrento et al. confirmed that multi-strain probiotic (*Bifidobacterium longum* subsp. *infantis* CECT 7210 and *Lactobacillus rhamnosus* HN001) with or without galacto-oligosaccharides fought against enterotoxigenic *Escherichia coli* F4 in an early weaned piglet model. Research of Wang Y. et al. showed that dietary *Lactobacillus plantarum* P8 supplementation improved the growth performance as well as the intestinal health of broilers infected with *Eimeria*. Wang T et al. and Astó et al. found probiotics had potential application to treat alcoholic liver disease induced by liquor and functional gastrointestinal disorders, respectively. Furthermore, the results from Ke et al. and Buddhasiri et al. showed the effects of probiotics in controlling pathogens infection, such as *Cronobacter* spp. and *Salmonella*. Other functions of probiotics have attracted more and more attention, such as the antifungal effect shown by Somashekaraiah et al. More and more researches showed that the marine active substances act beneficial effects on animals' health through the intestine. The modulating effect of astaxanthin on gut microbiota has been reported by Gao et al. in this Research Topic. These studies have expanded our understanding on this Research Topic; however, further studies are still needed to fully elucidate the underlying mechanisms.

## Author Contributions

All authors listed have made a substantial, direct, and intellectual contribution to the work and approved it for publication.

## Conflict of Interest

The authors declare that the research was conducted in the absence of any commercial or financial relationships that could be construed as a potential conflict of interest.

## Publisher's Note

All claims expressed in this article are solely those of the authors and do not necessarily represent those of their affiliated organizations, or those of the publisher, the editors and the reviewers. Any product that may be evaluated in this article, or claim that may be made by its manufacturer, is not guaranteed or endorsed by the publisher.
